# Supplementary material for: Scar matrix drives Piezo1 mediated stromal inflammation leading to placenta accreta spectrum
Source: Nat Commun. 2024 Sep 27;15:8379. doi: 10.1038/s41467-024-52351-0 (PMC11436960; doi:10.1038/s41467-024-52351-0)
Supplement: Supplementary file 1 — Supplementary Information [file 41467_2024_52351_MOESM1_ESM.pdf]

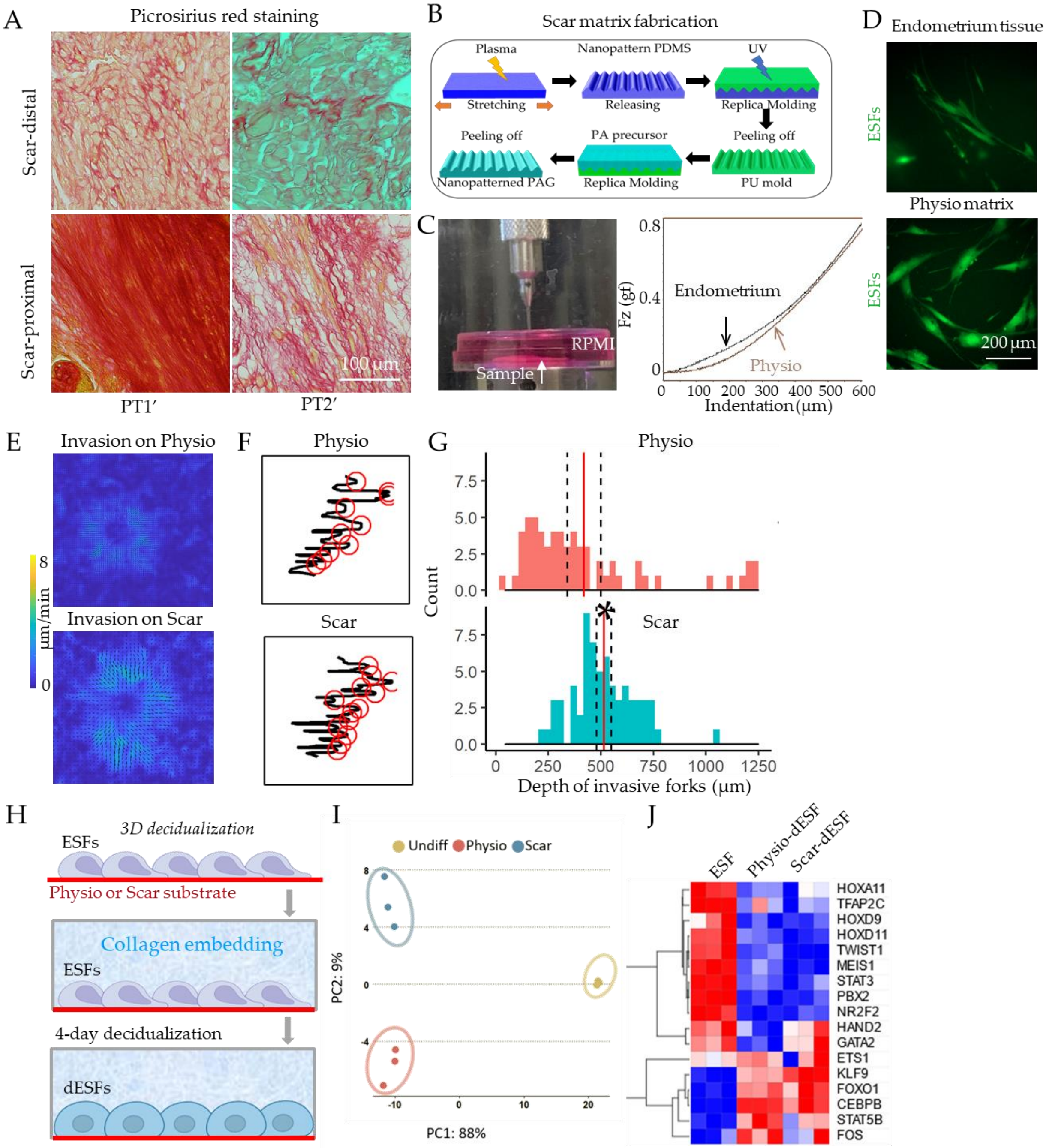

Supplementary Figure 1. (A) Picrosirius red staining of tissue sections from scar-proximal and scar-distal regions of PAS patients. n= 2 biological replicates with similar results. (B) Schematic showing the process of fabricating nanopatterned Scar matrix. (C) Photo of Mach-1 mechanical indenter measuring rigidity of endometrium sample in RPMI. Graph showing indentation force curves of endometrium and Physio matrix. (D) ESFs labeled with cell green tracker on endometrial tissue and Physio matrix. n= 2 biological replicates. (E) Mean velocity of HTR8 spheroids invaded into dESF monolayers on Physio and Scar over 24h. (F) Automated identification of the tip of the invading forks (red circles) in ANSIA. (G) Quantification for the

depth of invasion by each trophoblast fork invading into the dESFs. Solid red lines refer to the mean length of invasion, and black dashed lines refer to the 95% confidence intervals. *p = 0.03. (H) Schematic showing 3D decidualization process. ESFs are seeded on Physio and Scar. After attachment, ESFs were embedded in collagen type I. Afterwards, ESFs were treated with decidualization medium for 4 days. (I) PCA plot of RNA-seq from ESFs (Undiff) and dESFs on Physio and Scar. n= 3 biological replicates. (J) Decidualization related transcription factors expression levels in ESF, dESFs from Phyiso, and dESFs from Scar.

A

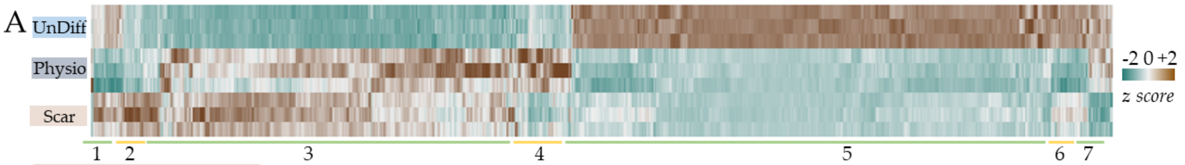

1. Scar >undiff >Physio
2. Scar > undiff ,Physio
3. Scar , Physio > undiff
4. Physio > Scar, undiff
5. Undiff > Scar, Physio
6. Undiff > Scar > Physio
7. Undiff , Physio > Scar

B Upregulated in Scar

Cluster 1 ID	Cluster 1 Description	-log10(p_adj)
GO:0044773	mitotic DNA damage checkpoint signaling	4.409
GO:0044774	mitotic DNA integrity checkpoint signaling	4.325
GO:0050727	regulation of inflammatory response	4.322
GO:0000077	DNA damage checkpoint signaling	3.959
GO:0001525	angiogenesis	3.909
GO:0031570	DNA integrity checkpoint signaling	3.834
GO:1902806	regulation of cell cycle G1/S phase transition	3.799
GO:0031347	regulation of defense response	3.749
GO:0071675	regulation of mononuclear cell migration	3.746
GO:0002685	regulation of leukocyte migration	3.743
R-HSA-1474244	Extracellular matrix organization	5.113
R-HSA-3000170	Syndecan interactions	4.438
hsa04210	Apoptosis	3.496
R-HSA-1474228	Degradation of the extracellular matrix	3.448
R-HSA-3000171	Non-integrin membrane-ECM interactions	3.416
R-HSA-76002	Platelet activation, signaling and aggregation	3.377
R-HSA-1442490	Collagen degradation	3.312
R-HSA-6798695	Neutrophil degranulation	2.988
R-HSA-216083	Integrin cell surface interactions	2.952
hsa04064	NF-kappa B signaling pathway	2.701

Cluster 2 ID	Cluster 2 Description	-log10(p_adj)
GO:0071345	cellular response to cytokine stimulus	11.604
GO:0032496	response to lipopolysaccharide	9.760
GO:0071396	cellular response to lipid	9.589
GO:0002237	response to molecule of bacterial origin	9.442
GO:0071222	cellular response to lipopolysaccharide	9.379
GO:0071219	cellular response to molecule of bacterial origin	9.132
GO:0071216	cellular response to biotic stimulus	8.629
GO:0009617	response to bacterium	8.434
GO:0006954	inflammatory response	8.336
GO:0045765	regulation of angiogenesis	8.189
R-HSA-449147	Signaling by Interleukins	10.071
R-HSA-6783783	Interleukin-10 signaling	10.062
R-HSA-1280215	Cytokine Signaling in Immune system	9.533
hsa05323	Rheumatoid arthritis	9.465
hsa04657	IL-17 signaling pathway	9.428
hsa04060	Cytokine-cytokine receptor interaction	8.923
hsa05417	Lipid and atherosclerosis	7.812
hsa04064	NF-kappa B signaling pathway	7.592
R-HSA-6785807	Interleukin-4 and Interleukin-13 signaling	7.478
hsa04668	TNF signaling pathway	7.368

Cluster 6 ID	Cluster 6 Description	-log10(p_adj)
GO:0060627	regulation of vesicle-mediated transport	3.872
GO:0007162	negative regulation of cell adhesion	3.645
GO:0032870	cellular response to hormone stimulus	3.375
GO:0034765	regulation of ion transmembrane transport	3.255
GO:0002040	sprouting angiogenesis	3.205
GO:0010812	negative regulation of cell-substrate adhesion	3.062
GO:0043535	regulation of blood vessel endothelial cell migration	3.062
GO:0008360	regulation of cell shape	3.041
GO:0001570	vasculogenesis	3.006
GO:0010001	glial cell differentiation	2.890
R-HSA-9012999	RHO GTPase cycle	4.401
R-HSA-1474228	Degradation of the extracellular matrix	3.174
R-HSA-2022090	Assembly of collagen fibrils and other multimeric structures	3.162
R-HSA-9658195	Leishmania infection	3.115
R-HSA-194315	Signaling by Rho GTPases	3.068
R-HSA-9716542	Signaling by Rho GTPases, Miro GTPases and RHOBTB3	3.005
R-HSA-1474244	Extracellular matrix organization	2.788
R-HSA-9013149	RAC1 GTPase cycle	2.727
R-HSA-388396	GPCR downstream signalling	2.684
R-HSA-1474290	Collagen formation	2.674

C Downregulated in Scar

Cluster 4 ID	Cluster 4 Description	-log10(p_adj)
GO:0001822	kidney development	7.332
GO:0072001	renal system development	7.196
GO:0001655	urogenital system development	6.669
GO:0072006	nephron development	5.868
GO:0032835	glomerulus development	5.414
GO:0009725	response to hormone	5.355
GO:0072210	metanephric nephron development	4.982
GO:0001656	metanephros development	4.585
GO:0045995	regulation of embryonic development	4.459
GO:0032870	cellular response to hormone stimulus	4.362
R-HSA-9013508	NOTCH3 Intracellular Domain Regulates Transcription	5.426
hsa04330	Notch signaling pathway	5.262
hsa05224	Breast cancer	4.379
R-HSA-9012852	Signaling by NOTCH3	4.236
hsa05200	Pathways in cancer	3.322
hsa00260	Glycine, serine and threonine metabolism	3.174
R-HSA-500792	GPCR ligand binding	3.036
R-HSA-211945	Phase I - Functionalization of compounds	2.948
R-HSA-2122947	NOTCH1 Intracellular Domain Regulates Transcription	2.941
R-HSA-71387	Metabolism of carbohydrates	

Cluster 7 ID	Cluster 7 Description	-log10(p_adj)
GO:0045598	regulation of fat cell differentiation	3.817
GO:0030199	collagen fibril organization	3.784
GO:0030239	myofibril assembly	3.673
GO:0055002	striated muscle cell development	3.652
GO:0001501	skeletal system development	3.427
GO:0007193	adenylate cyclase-inhibiting G protein-coupled receptor signaling pathway	3.425
GO:0014910	regulation of smooth muscle cell migration	3.264
GO:0097435	supramolecular fiber organization	3.236
GO:0010927	cellular component assembly involved in morphogenesis	2.994
GO:0031032	actomyosin structure organization	2.924
R-HSA-3560782	Diseases associated with glycosaminoglycan metabolism	4.190
R-HSA-3781865	Diseases of glycosylation	3.805
R-HSA-186797	Signaling by PDGF	3.738
R-HSA-2187338	Visual phototransduction	2.982
R-HSA-5668914	Diseases of metabolism	2.898
R-HSA-1630316	Glycosaminoglycan metabolism	2.774
R-HSA-71387	Metabolism of carbohydrates	2.630
R-HSA-196854	Metabolism of vitamins and cofactors	2.253
R-HSA-913531	Interferon Signaling	2.197

Supplementary Figure 2. (A) Hierarchical clustering of ESFs (Undiff) and dESFs on Physio, and dESFs on Scar. Compared to dESFs from Physio, dESFs from Scar shows upregulation in clusters 1, 2, and 6, and downregulation in clusters 4 and 7. n= 3 biological replicates. (B-C) Detail list of

the top 10 significant ontologies in each cluster upregulated or downregulated in Scar vs Physio dESFs.

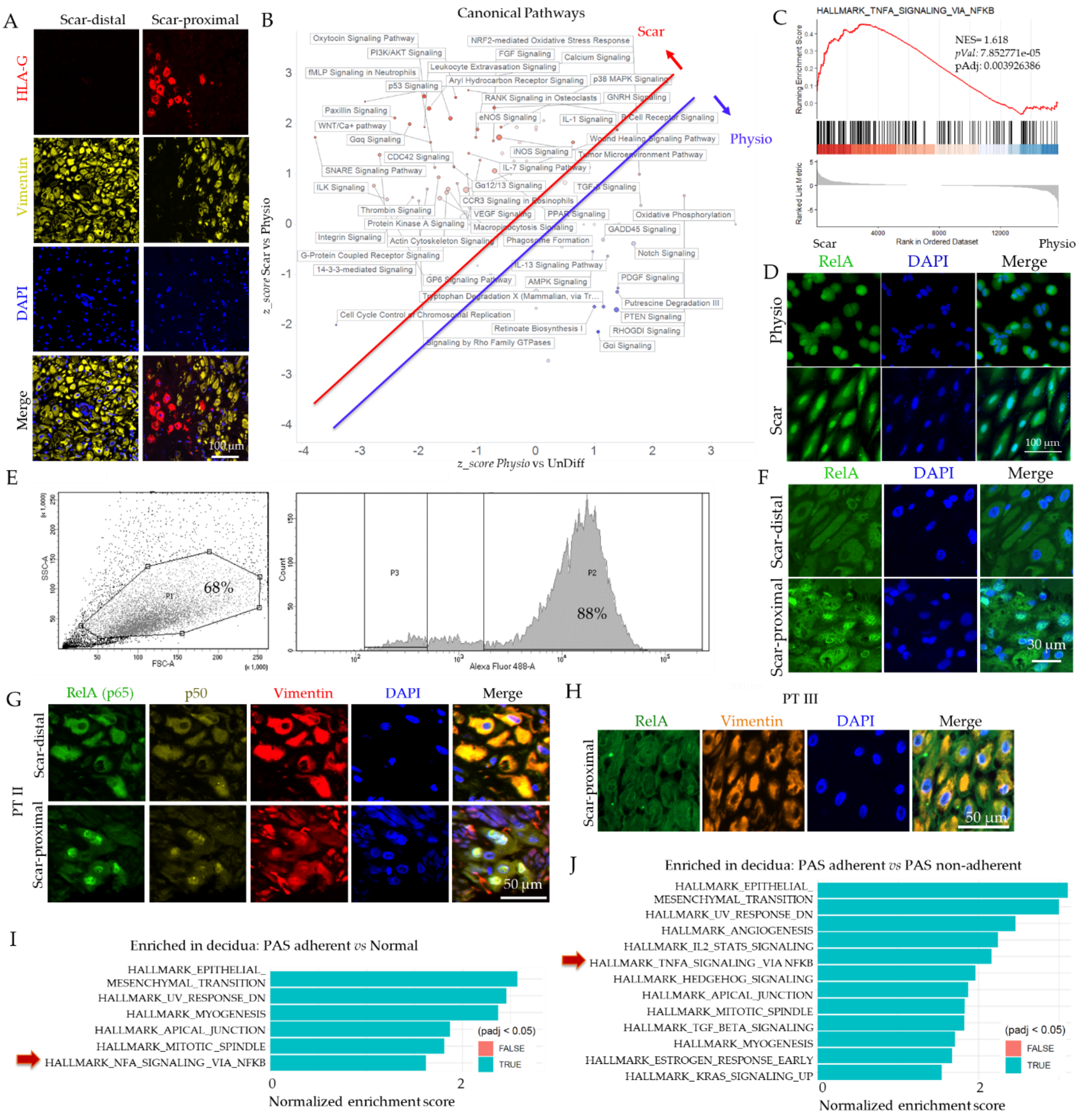

Supplementary Figure 3. (A) Immunocytochemistry staining of HLA-G, Vimentin, and DNA in scar-distal and scar-proximal regions of PAS tissue sections. n= 3 biological replicates. (B) Canonical pathway analysis of ESFs from Physio (Undiff), dESFs from Physio, and dESFs from Scar. (C) GSEA analysis of NFkB signaling pathway activation of dESFs from Scar and Physio. (D) Immunofluorescent staining of RelA (p65) and DNA in dESFs on Scar and Physio matrices. n= 3 biological replicates. (E) Flow sorting of HLA-G^{+} cells from cells isolated from decidua basalis. (F) Immunocytochemistry staining of RelA and DNA in scar-distal and scar-proximal regions of PAS tissue sections. n= 3 biological replicates. (G-H) Immunocytochemistry staining of RelA (p65), p50, Vimentin, and DNA in scar-distal and scar-proximal regions of PAS patients. n= 2 biological replicates. (I-J) GSEA pathway enrichment analysis of single-cell RNA sequencing

data from adherent and non-adherent decidua of PAS patients and decidua of normal pregnancy (GEO accession number GSE212505)[1]. Red arrows show that NFκB pathway is enriched in PAS adherent decidua compared to PAS normal decidua and (I) and non-adherent decidua (J).

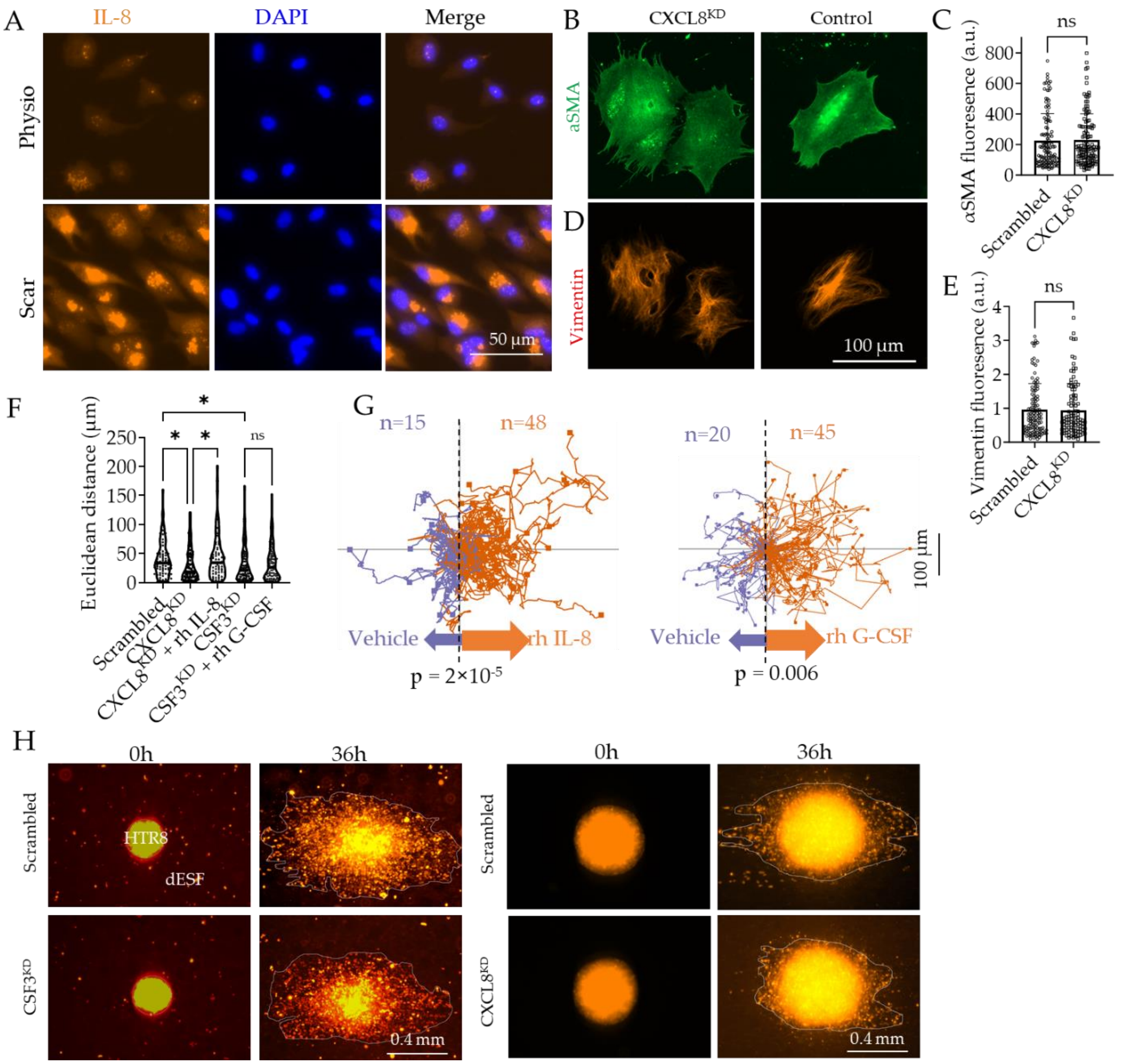

Supplementary Figure 4. (A) Immunofluorescent staining of IL-8 and DNA in dESFs on Scar and Physio after 4h of GolgiStop treatment. Immunofluorescent staining and intensity quantification of αSMA (B-C) and vimentin (D-E) in scrambled and CXCL8^{KD} dESFs. n=123 and 121 cells in (C); n=120 and 102 cells in (E). n= 3 biological replicates. (F) Euclidean distance of HTR8 migration conditioned with medium from scrambled, CXCL8^{KD}, and CSF3^{KD} dESFs. Corresponding 300 ng/mL recombinant IL-8 and G-CSF3 were added to the conditioned medium from CXCL8^{KD}, and CSF3^{KD} dESFs. n=86, 87, 71, 92, and 102 cells, respectively. p=0.0258, 0.0123, and 0.0238. (G) 3D chemotaxis trajectories of HTR8 trophoblasts in the gradients of recombinant IL-8 and G-CSF, respectively. P value show the power of chemoattraction using Rayleigh testing. (H) Fluorescent images showing HTR8 spheroid invaded into scrambled, CSF3^{KD}, and CXCL8^{KD} dESFs monolayer. n= 3 biological replicates. All bar graph data are shown as mean ± s.d.; Statistical significance is determined by unpaired two-tailed t-test (*$p<0.05$; ns: not significant).

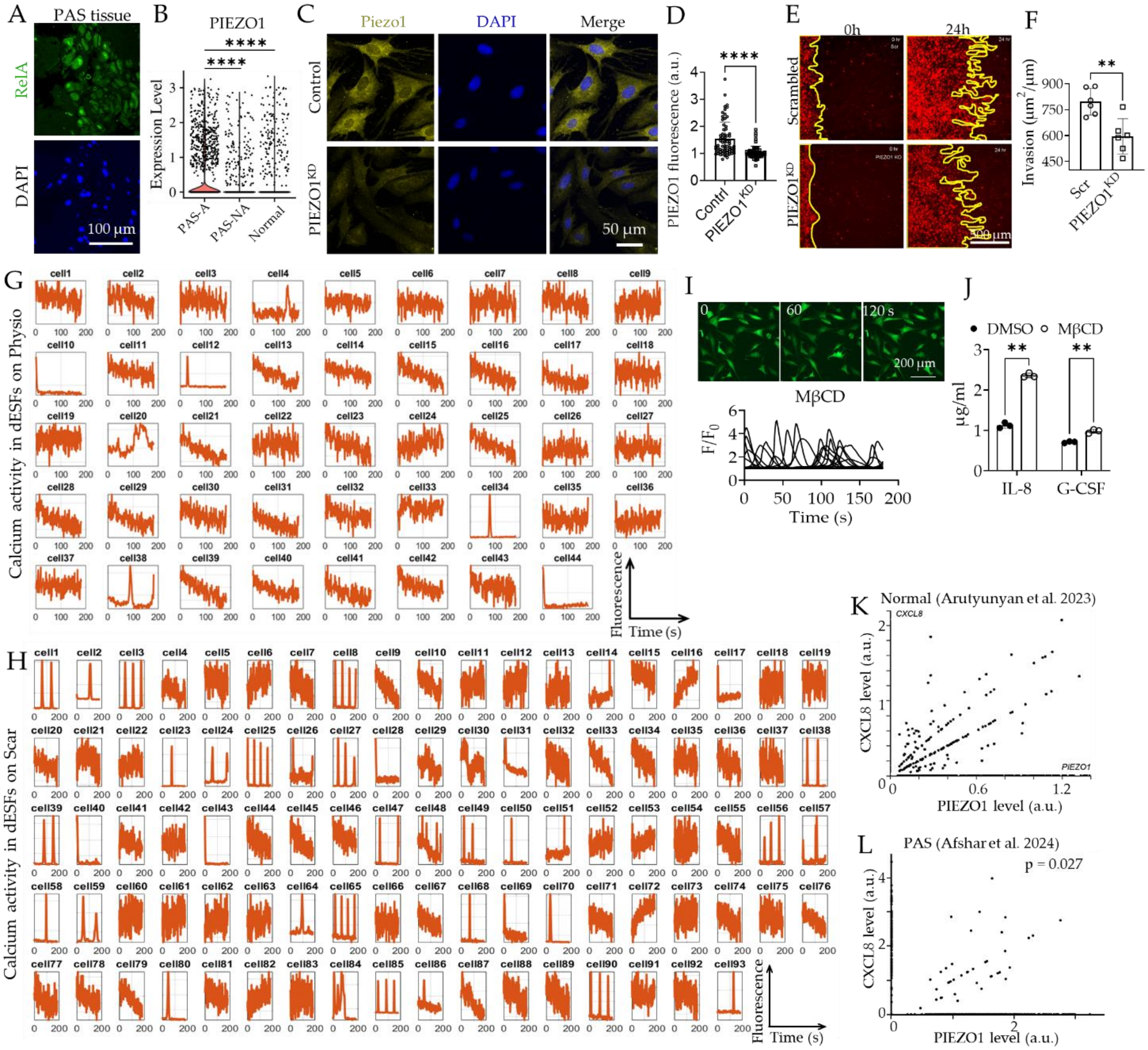

Supplementary Figure 5. (A) Immunohistochemistry staining of RelA and DNA on PAS tissue sections. n= 3 biological replicates. (B) PIEZO1 scRNA expression levels in adherent and non-adherent PAS decidua and normal decidua analyzed using GEO dataset GSE212505. n=1836, 1595, and 1246 cells; p=1.3×10^{-34} (PAS-A *vs* Normal) and 4.9×10^{-29}(PAS-A *vs* PAS-NA). (C) Specificity of Piezo1 antibody showed by staining of Piezo1 in dESFs and dESFs with PIEZO1 gene knockdown. Experiments are repeated twice with similar results. (D) Statistic analysis of Piezo1 protein fluorescence in (F). n=67 and 60 cells; p=4.7×10^{-8}. (E-F) Fluorescent images and bar graph showing HTR8-mCherry cells invaded into scrambled and PIEZO1^{KD} dESFs monolayer using ANSIA. n= 6 locations for each condition; p=0.0031. Experiments are repeated twice with similar results. (G-H) Intracellular calcium dynamics in dESFs with GCaMP6 calcium indicator on Physio (G) and Scar (H) matrices. Time-lapse videos were taken at 1Hz for a duration of 180s. Times are shown in X axis and fluorescent intensity are shown in Y axis. Each graph represents

an individual cell. (I) Time-lapse images and calcium transients showing intracellular Ca^{2+} activities in dESFs treated overnight with 1 mM Methyl-β-Cyclodextrins (MβCD); n = 32 cells. (J) Concentration of IL-8 and G-CSF in supernatant of control dESFs, and those silenced for PIEZO1 gene; as well as **(M)** dESFs treated overnight with 1mM MβCD. n=3 replicates for each condition; p=0.0029 and 0.0027. (K-L) Bivariate plots of gene expression of CXCL8 vs PIEZO1 in decidual stroma cells based on single-cell RNAseq data from normal pregnancy[2] (I), and single-cell RNAseq data from adherent decidua of PAS patients (GEO accession number GSE212505)[1] (J). All bar graph data are shown as mean ± s.d.; Statistical significance is determined by unpaired two-tailed t-test (**p<0.01 and ****p<0.0001; ns: not significant).

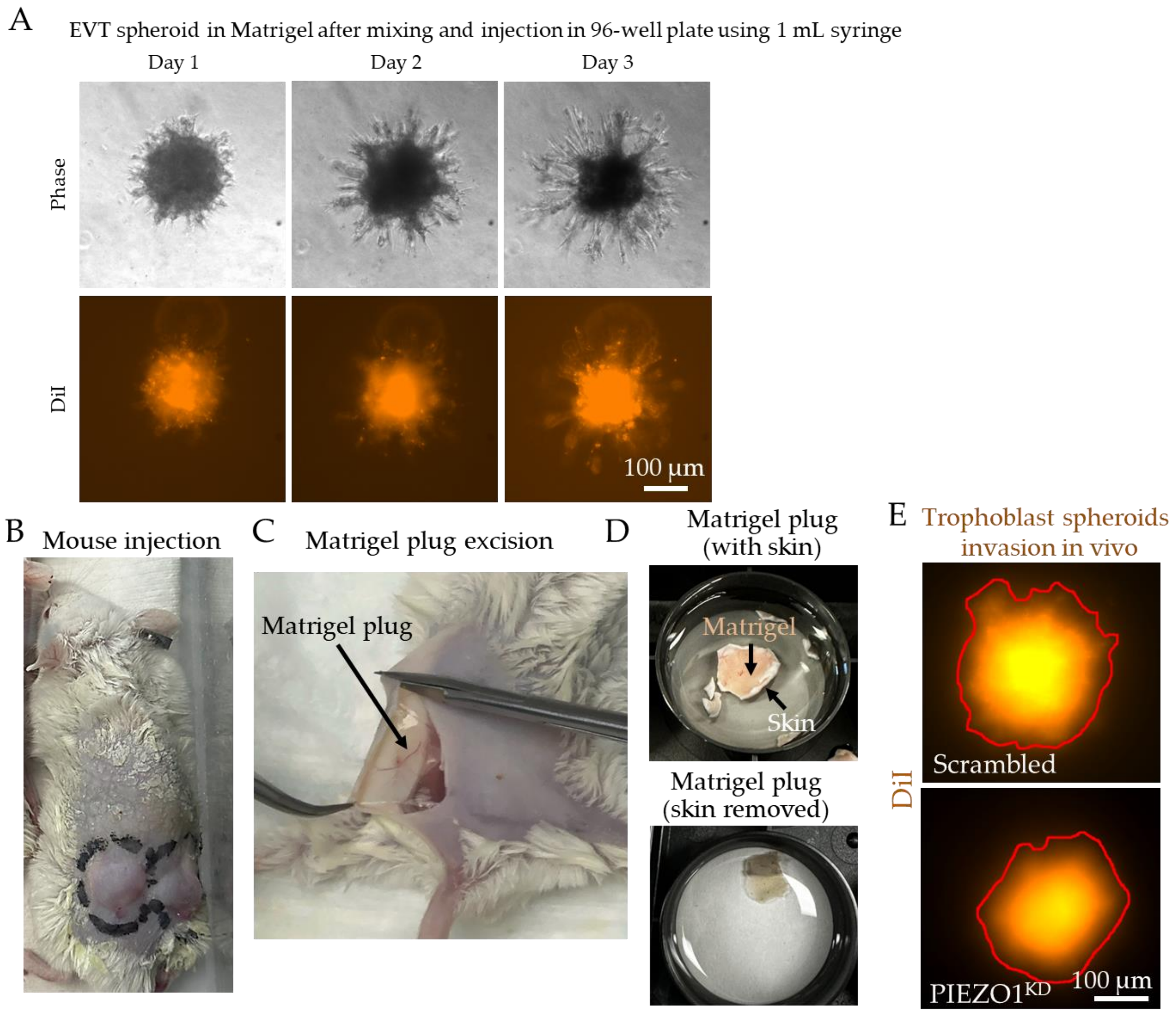

Supplementary Figure 6. (A) Images showing the integrity and capability to invasion of EVT spheroids in Matrigel in 96-well plate after the spheroids went through the same procedures for mouse injection. The experiment is one of the 9 biological replicates with similar results. (B) Representative photo of a mouse after injection of 0.2mL Matrigel containing EVT spheroids and dESFs. (C) Photo showing Matrigel plug excision. (D) Matrigel plug fixation in 4% PFA with and without skin. (E) Fluorescent images showing invasion of DiI labeled trophoblast spheroids into scrambled and PIEZO1^{KD} dESFs. The experiment is one of the 15 biological replicates with similar results.

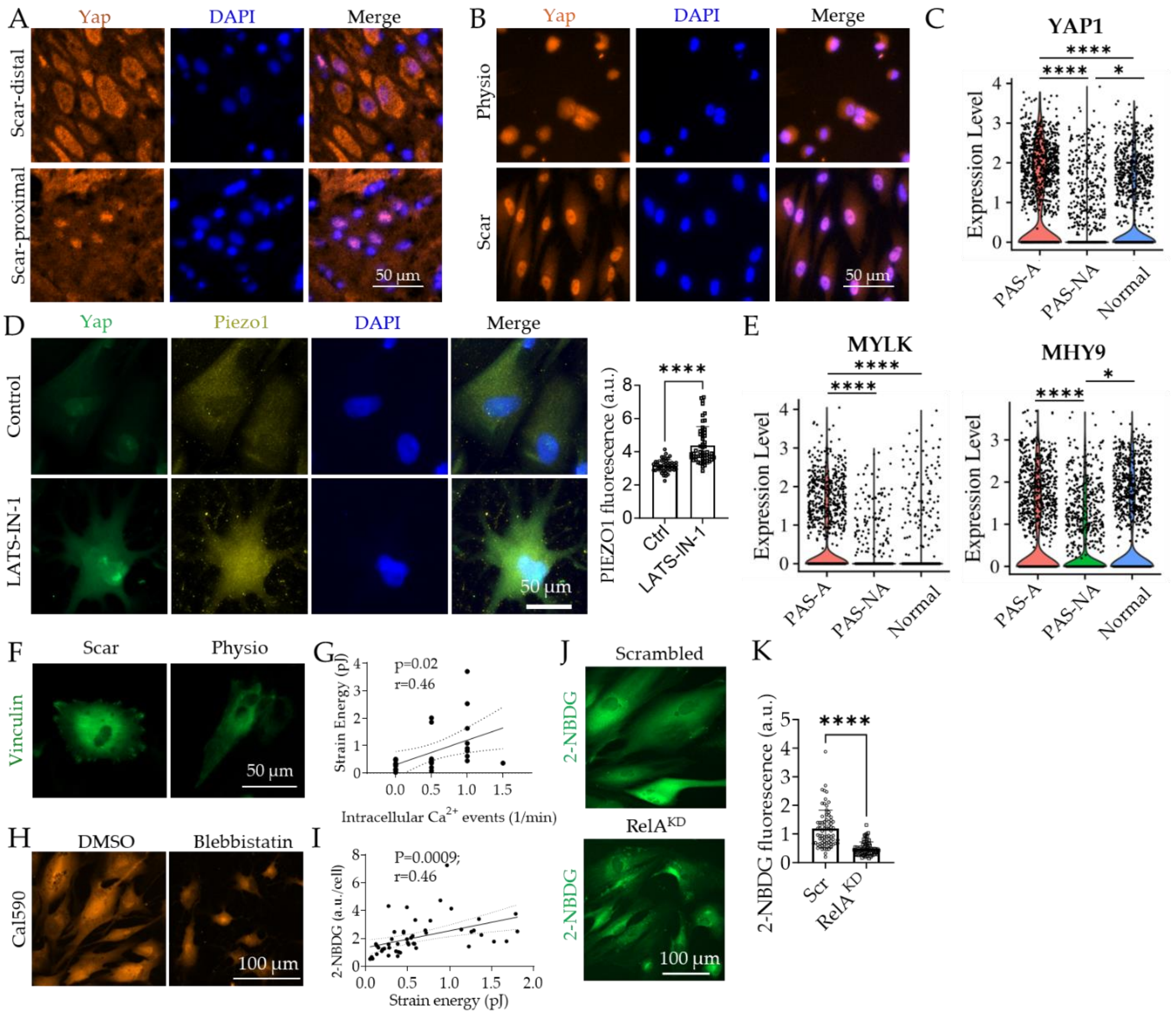

Supplementary Figure 7. Immunohistochemistry (A) and immunofluorescent (B) staining of Yap and DNA on PAS tissue sections (A) and dESFs on Physio and Scar matrices (B). Experiments are one of the three biological replicates with similar results. (C) YAP1 scRNA expression levels in adherent and non-adherent PAS decidua and normal decidua analyzed using GEO dataset GSE212505[1]. n=1836, 1595, and 1246 cells; $p=1.3\times10^{-63}$, 9.9×10^{-41}, and 1.2×10^{-7}. (D) Immunofluorescent staining of Yap, Piezo1, and DNA in dESFs treated with DMSO and 10μM LATS-IN-1 (Yap nuclear translocation promoter) for 24 hours. Graph shows statistical analysis of Piezo1 protein fluorescence. $p=1.9\times10^{-10}$. Experiments are repeated twice with similar results. (E) MYLK and MYH9 scRNA expression levels in adherent and non-adherent PAS decidua and normal decidua analyzed using GEO dataset GSE212505[1]. n=1836, 1595, and 1246 cells. $p=1.2\times10^{-77}$ and 5.8×10^{-102} (MYLK); $p=5.9\times10^{-11}$ and 6.1×10^{-7} (MYH9). (F) Vinculin expression in dESFs from Scar and Physio. Experiments are repeated twice with similar results. (G) Pearson correlation analysis of dESFs' contractile strain energy and intracellular calcium events frequency. r is Pearson correlation coefficient. (H) Fluorescent images shown calcium intensity of dESFs treated with DMSO and 30 μM Blebbistatin for 4h. Cells were loaded with Calbryte 590 to avoid the

autofluorescence of Blebbistatin. Experiments are repeated twice with similar results. (I) Pearson correlation analysis of dESFs' contractile strain energy and 2-NBDG uptake. r is Pearson correlation coefficient. (J) Fluorescent images of 2-NBDG uptake in scrambled and $RelA^{KD}$ dESFs. (K) Statistic analysis of 2-NBDG uptake. n=75 and 68 cells; $p=1.5\times10^{-13}$. Experiments are repeated twice with similar results. All bar graph data are shown as mean ± s.d.; Statistical significance is determined by unpaired two-tailed t-test (*$p<0.05$ and ****$p<0.0001$; ns: not significant).

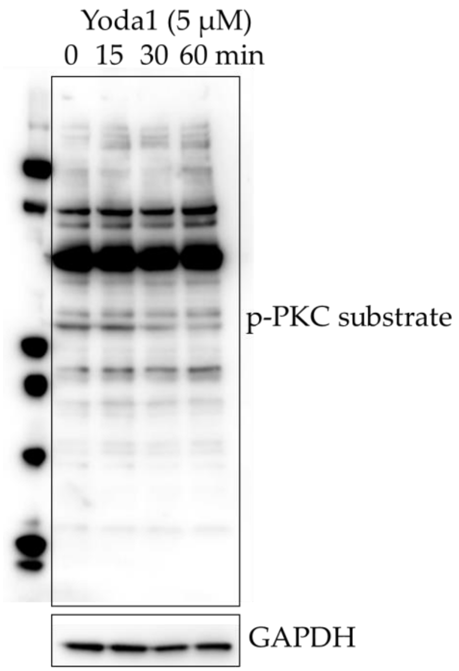

Supplementary Figure 8. Immunoblotting of p-PKC substrate in dESFs treated with 5 μM Yoda1 for 0, 15, 30, and 60 mins. The experiment was repeated 2 times with similar results.

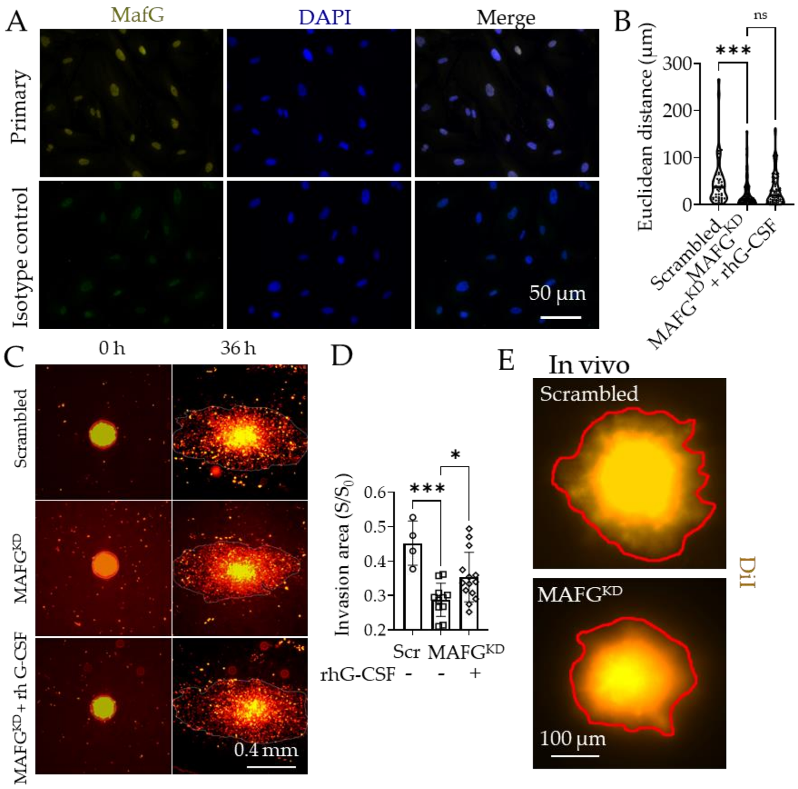

Supplementary Figure 9. (A) Fluorescent images of MafG and DNA in dESFs using rabbit primary antibody and rabbit IgG isotype control. (B) Euclidean distance of HTR8 migration in conditioned medium from scrambled and MAFG^{KD} dESFs, and MAFG^{KD} dESFs with the addition of rh G-CSF. n=44, 64, and 77 cells; p=0.0024 and 0.0888; ns: not significant. Experiments are repeated twice with similar results. (C) Fluorescent images showing HTR8-mCherry spheroid invaded into scrambled and MAFG^{KD} dESFs, and MAFG^{KD} dESFs with the addition of rh G-CSF. Experiments are repeated twice with similar results. (D) Invasion of HTR8 spheroids into scrambled and MAFG^{KD} dESFs, and MAFG^{KD} dESFs with the addition of rh G-CSF. n=4, 11, and 14 spheroids; p=0.001 and 0.0156. (E) Matrigel plug based in vivo invasion of DiI labeled HTR8 spheroids into surrounding scrambled and MAFG^{KD} dESFs in mouse. Experiments are one of the 16 biological replicates with similar results. All bar graph data are shown as mean ± s.d.; Statistical significance is determined by unpaired two-tailed t-test (*$p<0.05$ and ***$p<0.001$; ns: not significant).

Supplementary Table 1. Demographic information of PAS patients.

**Case number**	**Age/Years**	**Racial**	**Gender**
1	30-35	Caucasian	Female
2	30-35	Caucasian	Female
3	25-30	Black/African American	Female
4	30-35	Black/African American	Female
5	18-23	Caucasian	Female
6	25-30	Unknown	Female

Supplementary Table 2. Antibodies.

**Antibodies**	**Vendor**	**Cat. No.**	**Application**
Piezo1 (extracellular domain)	Proteintech	15939-1-AP	IF, 1:100
IL-8	Proteintech	27095-1-AP	IF, 1:100
G-CSF	Proteintech	17185-1-AP	IF, 1:100
MafG	GeneTex	GTX114541	IF, 1:100<br>WB, 1:1000
Phospho-NF-κB p65 (Ser536)	Cell Signaling Technology	3033S	WB, 1:1000
NF-κB p65 Polyclonal antibody	Proteintech	10745-1-AP	IF, 1:100<br>WB, 1:1000
NFKB1, p105, p50 Polyclonal antibody	Proteintech	14220-1-AP	IF, 1:100
Alexa Fluor 488 anti-human HLA-G Antibody	BioLegend	335918	IF, 1:100<br>Flow, 1:50
HLA-G Monoclonal antibody	Proteintech	16913-1-AP	IF, 1:100
Alexa Fluor 594 anti-Vimentin Antibody	BioLegend	677804	IF, 1:100
Yap	Cell Signaling Technology	12395s	IF, 1:100
Phospho-PKC Substrate Motif	Cell Signaling Technology	6967s	WB, 1:1000
GAPDH	Proteintech	60004-1-Ig	WB, 1:1000
NRF2, NFE2L2	Proteintech	66504-1-Ig	WB, 1:1000
Rabbit IgG control Polyclonal antibody	Proteintech	30000-0-AP	IF, 1:100
Alexa Fluor 488 Goat Anti-Mouse IgG(H+L)	Invitrogen	A32723	IF, 1:400
Alexa Fluor 594 Goat Anti-Rabbit IgG(H+L)	Invitrogen	A11012	IF, 1:400
Alexa Fluor 647 Goat Anti-Rabbit IgG(H+L)	Invitrogen	A32733	IF, 1:400

1. Afshar, Y. *et al.* Placenta accreta spectrum disorder at single-cell resolution: a loss of boundary limits in the decidua and endothelium. *Am. J. Obstet. Gynecol.* S0002-9378(23)00729-9. 29 (2024)

2. Arutyunyan, A. *et al.* Spatial multiomics map of trophoblast development in early pregnancy. *Nature* **616**, 143–151 (2023).

3. Vento-Tormo, R. *et al.* Single-cell reconstruction of the early maternal–fetal interface in humans. *Nature* **563**, 347–353 (2018).
