# Supplementary material for: Scar matrix drives Piezo1 mediated stromal inflammation leading to placenta accreta spectrum
Source: Nat Commun. 2024 Sep 27;15:8379. doi: 10.1038/s41467-024-52351-0 (PMC11436960; doi:10.1038/s41467-024-52351-0)
Supplement: Supplementary file 3 — Description Of Additional Supplementary File [file 41467_2024_52351_MOESM3_ESM.pdf]

Description of Additional supplementary file

Supplementary_Movie1: Phase contrast movie showing in-situ HTR8 spheroids invasion into dESF monolayers on Physio and Scar. Timelapse images were taken using Zeiss Axio Observer Z1 microscope and videos were.

Supplementary_Movie2: Fluorescent movie showing in -situ HTR8 spheroids (labeled with Cell Tracker Green) invasion into dESF monolayers on Physio and Scar. Corresponding to Figure 1I

Supplementary_Movie3: HTR8 -mCherry monolayers invaded into dESF monolayers detached from Physio and Scar, respectively. HTR8 were stably transduced with plasmid expressing H2B - bound mCherry driven by CMV promoter. Masks showing the invasive fronts of HTR8. Corresponding to Figure 1J .

Supplementary_Movie4: Green tracker labeled primary EVT spheroid invaded into NFKB1KD dESF monolayer on nanopatterned surface. Time -lapse videos were taken every 2 hours. Corresponding to Figure 2F .

Supplementary_Movie5: HTR8 -mCherry migration (left) and the corresponding single -cell tracking of HTR8 nuclear (right) using TrackMate plugin in Fiji/ImageJ. Curves marked cell trajectories. Corresponding to Figure 3G.

Supplementary_Movie6: Primary EVT chemotaxis in a 3D chemotaxis chamber (Ibidi). Primary EVT were encapsulated in 1mg/ml collagen type I loaded into a microchannel with a width of 1mm and a height of 70 µm. Human recombinant protein IL -8 were loaded to the right side of the microchannel at the concentration of 300ng/ml. Cell nuclear were tracked using TrackMate plugin in Fiji/ImageJ. Corresponding to Figure 3I

Supplementary_Movie7: Green tracker labeled primary EVT spheroid invaded into PIEZO1KD dESF monolayer on nanopatterned surface. Time -lapse videos were taken every 2 hours. Corresponding to Figure 4C

Supplementary_Movie8: Calcium activities in genetic calcium indicator GCamp6f transduced dESFs on Physio and Scar. Time -lapse images were taken using Zeiss Axio Observer Z1 microscope and videos were created using ImageJ. Corresponding to Figure 4F

Supplementary_Movie9: Calcium activities in dESFs before and after 3µM Yoda1 treatment. Cells were preloaded with 2µM Fluo4-AM. Corresponding to Figure 4I.

Supplementary_Movie10: Calcium activities in dESFs on a TFM gel with a stiffness of 6 kPa. Cells were preloaded with 2µM Fluo4-AM. Cells were detached immediately after Calcium imaging for the following gel displacement measurement. Corresponding to Figure 5F

Supplementary_Movie11: Calcium activities in dESFs with DMSO and 30µM Blebbistatin treatment for 2h. Cells were loaded with 1µM Calbryte 590 after treatment. Corresponding to Figure 5H.

Supplementary_Movie12: Green tracker labeled primary EVT spheroid invaded into MAFGKD dESF monolayer on nanopatterned surface. Time-lapse videos were taken every 2 hours. Corresponding to Figure 7J.
