# Supplementary material for: Scar matrix drives Piezo1 mediated stromal inflammation leading to placenta accreta spectrum
Source: Nat Commun. 2024 Sep 27;15:8379. doi: 10.1038/s41467-024-52351-0 (PMC11436960; doi:10.1038/s41467-024-52351-0)
Supplement: Supplementary file 16 — Reporting summary [file 41467_2024_52351_MOESM16_ESM.pdf]

nature portfolio

Corresponding author(s): Kshitiz

Last updated by author(s): Jun 4, 2024

Reporting Summary

Nature Portfolio wishes to improve the reproducibility of the work that we publish. This form provides structure for consistency and transparency in reporting. For further information on Nature Portfolio policies, see our Editorial Policies and the Editorial Policy Checklist.

Statistics

For all statistical analyses, confirm that the following items are present in the figure legend, table legend, main text, or Methods section.

n/a	Confirmed	
☐	☒	The exact sample size (*n*) for each experimental group/condition, given as a discrete number and unit of measurement
☐	☒	A statement on whether measurements were taken from distinct samples or whether the same sample was measured repeatedly
☐	☒	The statistical test(s) used AND whether they are one- or two-sided<br>*Only common tests should be described solely by name; describe more complex techniques in the Methods section.*
☒	☐	A description of all covariates tested
☒	☐	A description of any assumptions or corrections, such as tests of normality and adjustment for multiple comparisons
☐	☒	A full description of the statistical parameters including central tendency (e.g. means) or other basic estimates (e.g. regression coefficient) AND variation (e.g. standard deviation) or associated estimates of uncertainty (e.g. confidence intervals)
☐	☒	For null hypothesis testing, the test statistic (e.g. *F*, *t*, *r*) with confidence intervals, effect sizes, degrees of freedom and *P* value noted<br>*Give P values as exact values whenever suitable.*
☒	☐	For Bayesian analysis, information on the choice of priors and Markov chain Monte Carlo settings
☒	☐	For hierarchical and complex designs, identification of the appropriate level for tests and full reporting of outcomes
☐	☒	Estimates of effect sizes (e.g. Cohen's *d*, Pearson's *r*), indicating how they were calculated

Our web collection on statistics for biologists contains articles on many of the points above.

Software and code

Policy information about availability of computer code

Data collection	Fluorescent, Apotome, and time-lapse images were collected by Zeiss Observer Z1 microscope (Zen Blue 2.6 Pro) with PECON Incubation System.<br>Whole slide scanning was collected using Zeiss Axioscan 7 Microscope Slide Scanner.<br>Tissue rigidity was collected by Mach-1 micromechanical testing system (Biomomentum).<br>Matrix surface nanotopography was collected by Cypher ES Environmental AFM.<br>RNAseq was evaluated with Bioanalyzer 2100 (Agilent).<br>Immunoblots were collected by Imager (Molecular Biosciences).<br>Cells sorting was collected by BECTON-DICKINSON FACSARIA II sorter.<br>CRISPR/Cas9 for in vivo experiments was performed using Neon NxT Electroporation System (Invitrogen).<br>Oxidative phosphorylation and glycolysis were collected by Seahorse XF analyzer (Agilent Technologies).<br>ELISA signals were collected by SpectraMax i3x microplate reader (Molecular Devices).
Data analysis	Data were analyzed using PRISM 9. Two-tailed unpaired Student’s t-test was implemented for statistical significance of the differences between two groups. For graphs showing correlation analysis, Pearson correlation coefficients were provided together with two-tailed statistical significance values.<br>In-situ cell migration was analyzed using Matlab 2023a PIVlab.<br>Calcium dynamics were analyzed using ImageJ and Prism 9.<br>Directional cell migration was analyzed using Chemotaxis and Migration Tool V2.0 (ibidi) and TrackMate plugin in Fiji/ImageJ.<br>Traction force was quantified using pyTFM 1.1.

For manuscripts utilizing custom algorithms or software that are central to the research but not yet described in published literature, software must be made available to editors and reviewers. We strongly encourage code deposition in a community repository (e.g. GitHub). See the Nature Portfolio guidelines for submitting code & software for further information.

Data

Policy information about availability of data

All manuscripts must include a data availability statement. This statement should provide the following information, where applicable:

- Accession codes, unique identifiers, or web links for publicly available datasets
- A description of any restrictions on data availability
- For clinical datasets or third party data, please ensure that the statement adheres to our policy

The publicly available single cell sequencing data used in this study are available in the GEO database with GEO accession number of GSE212505. Source data are provided with this paper.

Research involving human participants, their data, or biological material

Policy information about studies with human participants or human data. See also policy information about sex, gender (identity/presentation), and sexual orientation and race, ethnicity and racism.

Reporting on sex and gender	This article focuses on abnormal placentation during preganancy, therefore biospy are all collected from deidentified female patients.
Reporting on race, ethnicity, or other socially relevant groupings	Not applicable
Population characteristics	Fertile female
Recruitment	Patients with placenta accreta spectrum were identified by UConn John Dempsey Hospital or clinical partners of Accio Biobank Online.
Ethics oversight	Human endometrial and PAS specimens were provided by University of Connecticut Health Center after de-identification, or by Accio Biobank Online with IRB waivor.

Note that full information on the approval of the study protocol must also be provided in the manuscript.

Field-specific reporting

Please select the one below that is the best fit for your research. If you are not sure, read the appropriate sections before making your selection.

☒ Life sciences ☐ Behavioural & social sciences ☐ Ecological, evolutionary & environmental sciences

For a reference copy of the document with all sections, see nature.com/documents/nr-reporting-summary-flat.pdf

Life sciences study design

All studies must disclose on these points even when the disclosure is negative.

Sample size	No method was used to predetermine sample size.
Data exclusions	No data was excluded.
Replication	All experiments were performed with at least three biological replicates with similar results.
Randomization	Mice were randomly divided into different groups. For in vitro assays when relevant, fields of view or cells were randomly chosen for analysis.
Blinding	Mice data were analyzed blindly. In vitro data were analyzed blindly using softwares with the same experiment-dependent thresholdings/ setups.

Reporting for specific materials, systems and methods

We require information from authors about some types of materials, experimental systems and methods used in many studies. Here, indicate whether each material, system or method listed is relevant to your study. If you are not sure if a list item applies to your research, read the appropriate section before selecting a response.

Materials & experimental systems

n/a	Involved in the study
☐	☒ Antibodies
☐	☒ Eukaryotic cell lines
☒	☐ Palaeontology and archaeology
☐	☒ Animals and other organisms
☒	☐ Clinical data
☒	☐ Dual use research of concern
☒	☐ Plants

Methods

n/a	Involved in the study
☒	☐ ChIP-seq
☐	☒ Flow cytometry
☒	☐ MRI-based neuroimaging

Antibodies

Antibodies used

Antibodies/ Vendor/ Cat. No.
Piezo1 (extracellular domain)/ Proteintech/ 15939-1-AP
IL-8/ Proteintech/ 27095-1-AP
G-CSF/ Proteintech/ 17185-1-AP
MafG/ GeneTex/ GTX114541
Phospho-NF-κB p65 (Ser536)/ Cell Signaling Technology/ 3033S
NF-κB p65 Polyclonal antibody/ Proteintech/ 10745-1-AP
NFKB1,p105,p50 Polyclonal antibody/ Proteintech/ 14220-1-AP
Alexa Fluor 488 anti-human HLA-G Antibody/ BioLegend/ 335918
HLA-G Monoclonal antibody/ Proteintech/ 16913-1-AP
Alexa Fluor 594 anti-Vimentin Antibody/ BioLegend/ 677804
Yap/ Cell Signaling Technology/ 12395s
Phospho-PKC Substrate Motif/ Cell Signaling Technology/ 6967s
GAPDH/ Proteintech/ 60004-1-Ig
NRF2, NFE2L2/ Proteintech/ 66504-1-Ig
Rabbit IgG control Polyclonal antibody/ Proteintech/ 30000-0-AP
Alexa Fluor 488 Goat Anti-Mouse IgG(H+L)/ Invitrogen/ A32723
Alexa Fluor 594 Goat Anti-Rabbit IgG(H+L)/ Invitrogen/ A11012
Alexa Fluor 647 Goat Anti-Rabbit IgG(H+L)/ Invitrogen/ A32733

Validation

Piezo1 (extracellular domain)/ Proteintech/ 15939-1-AP: https://www.ptglab.com/products/pictures/pdf/15939-1-AP.pdf
IL-8/ Proteintech/ 27095-1-AP: https://www.ptglab.com/Products/Pictures/pdf/27095-1-AP.pdf
G-CSF/ Proteintech/ 17185-1-AP: https://www.ptglab.com/Products/Pictures/pdf/17185-1-AP.pdf
MafG/ GeneTex/ GTX114541: https://www.genetex.com/PDF/Download?catno=GTX114541
Phospho-NF-κB p65 (Ser536)/ Cell Signaling Technology/ 3033S: https://www.cellsignal.com/datasheet.jsp?productId=3033&images=1
NF-κB p65 Polyclonal antibody/ Proteintech/ 10745-1-AP: https://www.ptglab.com/Products/Pictures/pdf/10745-1-AP.pdf
NFKB1,p105,p50 Polyclonal antibody/ Proteintech/ 14220-1-AP: https://www.ptglab.com/Products/Pictures/pdf/14220-1-AP.pdf
Alexa Fluor 488 anti-human HLA-G Antibody/ BioLegend/ 335918: https://d1spbj2x7qk4bg.cloudfront.net/de-at/products/alexa-fluor-488-anti-human-hla-g-antibody-15424?pdf=true&displayInline=true&leftRightMargin=15&topBottomMargin=15&filename=Alexa%20Fluor%C2%AE%20488%20anti-human%20HLA-G%20Antibody.pdf&v=20240410063626
HLA-G Monoclonal antibody/ Proteintech/ 16913-1-AP: https://www.ptglab.com/products/pictures/pdf/16913-1-AP.pdf
Alexa Fluor 594 anti-Vimentin Antibody/ BioLegend/ 677804: https://www.biolegend.com/en-us/products/alexa-fluor-594-anti-vimentin-antibody-12146?pdf=true&displayInline=true&leftRightMargin=15&topBottomMargin=15&filename=Alexa%20Fluor%C2%AE%20594%20anti-Vimentin%20Antibody.pdf&v=20240501052741
Yap/ Cell Signaling Technology/ 12395s: https://www.cellsignal.com/datasheet.jsp?productId=12395&images=1
Phospho-PKC Substrate Motif/ Cell Signaling Technology/ 6967s: https://www.cellsignal.com/datasheet.jsp?productId=6967&images=1
GAPDH/ Proteintech/ 60004-1-Ig: https://ptglab.com/Products/Pictures/pdf/60004-1-Ig.pdf
NRF2, NFE2L2/ Proteintech/ 66504-1-Ig: https://ptglab.com/Products/Pictures/pdf/66504-1-Ig.pdf
Rabbit IgG control Polyclonal antibody/ Proteintech/ 30000-0-AP: https://www.ptglab.com/products/pictures/pdf/30000-0-AP.pdf
Alexa Fluor 488 Goat Anti-Mouse IgG(H+L)/ Invitrogen/ A32723: https://www.thermofisher.com/order/genome-database/dataSheetPdf?producttype=antibody&productsubtype=antibody_secondary&productId=A32723&version=Local
Alexa Fluor 594 Goat Anti-Rabbit IgG(H+L)/ Invitrogen/ A11012: https://www.thermofisher.com/order/genome-database/dataSheetPdf?producttype=antibody&productsubtype=antibody_secondary&productId=A-11012&version=Local
Alexa Fluor 647 Goat Anti-Rabbit IgG(H+L)/ Invitrogen/ A32733: https://www.thermofisher.com/order/genome-database/dataSheetPdf?producttype=antibody&productsubtype=antibody_secondary&productId=A32733&version=Local

Eukaryotic cell lines

Policy information about cell lines and Sex and Gender in Research

Cell line source(s)

HTR8/SVneo was purchased from ATCC (CRL-3271); Endometrial stromal fibroblasts and EVTs were isolated from patients biospy collection at UConn Health.

Authentication

STR

Mycoplasma contamination	Negtive
Commonly misidentified lines (See ICLAC register)	No misidentified lines used.

Animals and other research organisms

Policy information about studies involving animals; ARRIVE guidelines recommended for reporting animal research, and Sex and Gender in Research

Laboratory animals	Three months old SCID/beige male mice were purchased from Inotiv. Mice were kept in a 12 h light–dark cycle, temperature-controlled (22 ± 2 °C) and humidity-controlled (55 ± 5%) environment and fed a standard chow diet.
Wild animals	No wild animals are used in this study.
Reporting on sex	Only male mice were used in this study to eliminate the potential bias caused by the variants of progesterones in female mice.
Field-collected samples	No filed-collected samples are used in this study.
Ethics oversight	All animal protocols were approved by the Institutional Animal Care and Use Committee (IACUC) at the University of Connecticut Health Center before study initiation. All experiments were performed in accordance with IACUC guidelines, and abides by the ARRIVE guidelines for reporting animal experiments.

Note that full information on the approval of the study protocol must also be provided in the manuscript.

Plants

Seed stocks	*Report on the source of all seed stocks or other plant material used. If applicable, state the seed stock centre and catalogue number. If plant specimens were collected from the field, describe the collection location, date and sampling procedures.*
Novel plant genotypes	*Describe the methods by which all novel plant genotypes were produced. This includes those generated by transgenic approaches, gene editing, chemical/radiation-based mutagenesis and hybridization. For transgenic lines, describe the transformation method, the number of independent lines analyzed and the generation upon which experiments were performed. For gene-edited lines, describe the editor used, the endogenous sequence targeted for editing, the targeting guide RNA sequence (if applicable) and how the editor was applied.*
Authentication	*Describe any authentication procedures for each seed stock used or novel genotype generated. Describe any experiments used to assess the effect of a mutation and, where applicable, how potential secondary effects (e.g. second site T-DNA insertions, mosiacism, off-target gene editing) were examined.*

Flow Cytometry

Plots

Confirm that:

☒ The axis labels state the marker and fluorochrome used (e.g. CD4-FITC).

☒ The axis scales are clearly visible. Include numbers along axes only for bottom left plot of group (a 'group' is an analysis of identical markers).

☒ All plots are contour plots with outliers or pseudocolor plots.

☒ A numerical value for number of cells or percentage (with statistics) is provided.

Methodology

Sample preparation	Cells isolated from termed placenta were labeled with Alexa Fluor 488 conjugated HLA-G antibody in the dark. Cells are then washed with PBS and resuspended in PBS before flow sorting. HTR8-mCherry were prepared without labeling.
Instrument	BECTON-DICKINSON FACSARIA II sorter.
Software	BD FACSDiva Software
Cell population abundance	Around 80%
Gating strategy	Cells were gated using FSC-A/SSC-A to remove debris. Then, cells were gated by Alexa Fluor 488-A to get HLA-G positive cells; In another experiment, gated by mCherry to get HTR8 mCherry cells.

☒ Tick this box to confirm that a figure exemplifying the gating strategy is provided in the Supplementary Information.
